# Association Between the Bone Density of Alveolar and General Skeletal Bones in the Young

**DOI:** 10.7759/cureus.78643

**Published:** 2025-02-06

**Authors:** Noriyuki Sugino, Hiroko Kuroiwa, Hizuru Osanai, Shinichiro Yamada, Nanae Dewake, Souhei Suzuki, Yoshimi Kawahara, Nobuo Yoshinari, Nobuyuki Udagawa, Akira Taguchi

**Affiliations:** 1 Department of Oral and Maxillofacial Radiology, School of Dentistry, Matsumoto Dental University, Shiojiri, JPN; 2 Department of Pediatric Dentistry, School of Dentistry, Matsumoto Dental University, Shiojiri, JPN; 3 Department of Operative Dentistry, Endodontology and Periodontology, School of Dentistry, Matsumoto Dental University, Shiojiri, JPN; 4 Department of Hard Tissue Research, Graduate School of Oral Medicine, Matsumoto Dental University, Shiojiri, JPN; 5 Department of Orthodontics, School of Dentistry, Matsumoto Dental University, Shiojiri, JPN; 6 Department of Biochemistry, School of Dentistry, Matsumoto Dental University, Shiojiri, JPN

**Keywords:** alveolar bone mineral density, bone mineral density (bmd), general skeletal bone mineral density, intraoral radiographs, osteoporosis screening

## Abstract

Objective

Osteoporosis-related fractures are a significant health issue in aging societies, necessitating effective screening and prevention strategies. While panoramic radiographs are widely used for osteoporosis screening via mandibular cortical bone morphology, there is insufficient consensus on the quantitative analysis of alveolar bone mineral density (al-BMD) using intraoral radiographs. This study aimed to measure al-BMD in young adults and investigate its relationship with general skeletal bone mineral density (gs-BMD) at the lumbar spine (LSBMD) and femoral neck (FNBMD). Additionally, the influence of biological factors, such as body weight and height, on BMD was assessed.

Methodology

A total of 53 young adults (34 males, 19 females, mean age: 26.0 years) participated in this study. Intraoral radiographs with calcium carbonate reference standards quantified al-BMD at three mandibular premolar regions. gs-BMD was measured using dual-energy X-ray absorptiometry (DXA). Correlations among al-BMD, gs-BMD, and biological factors were analyzed.

Results

Results showed no significant differences between age, sex, body height, body weight, al-BMD, and gs-BMD. Age was correlated with FNBMD but not al-BMD as well as LSBMD. Body weight positively correlated with LSBMD (r = 0.437, P = 0.001) but negatively with FNBMD (r = -0.412, P = 0.002). No significant correlations were observed between al-BMD and gs-BMD or biological factors.

Conclusion

These findings suggest that al-BMD may not necessarily reflect gs-BMD changes and is strongly influenced by local factors in the oral cavity. This highlights the importance of evaluating al-BMD independently of gs-BMD. Future studies with larger sample sizes and additional factors influencing al-BMD are needed to validate these results.

## Introduction

Currently, the increase in mortality rates and medical costs due to fractures caused by osteoporosis has become a significant issue. Particularly in aging societies, osteoporosis-related fractures represent a severe health issue. Their prevention and early detection are critically important. In the field of dentistry, the classification of mandibular cortical bone morphology using panoramic radiographs has been recognized as a useful method for osteoporosis screening [1,2], and it has been included in clinical guidelines [3]. This method is relatively simple, non-invasive, and has become a widely accepted tool in routine dental practice.

On the other hand, there is insufficient consensus regarding the quantitative analysis of alveolar bone mineral density (al-BMD) using intraoral radiographs, despite their widespread use in dental practice due to lower radiation exposure and greater safety compared to panoramic radiographs. While al-BMD is strongly influenced by local factors in the oral cavity, such as inflammation, occlusal forces, and periodontal disease [4], its relationship with general skeletal bone mineral density (gs-BMD) has not been fully elucidated.

Therefore, this study aimed to measure al-BMD using intraoral radiographs in young adults and investigate its relationship with gs-BMD (lumbar spine and femur neck). Young adults were selected as the target population because their bone mineral density (BMD) is generally at or near its peak during this period [5]. This minimizes the influence of aging and allows for a clearer investigation into the relationship between al-BMD and gs-BMD. Additionally, this study examined the impact of biological factors, such as body weight and body height, on both al-BMD and gs-BMD to elucidate the unique characteristics of each site.

## Materials and methods

The participants of this study consisted of 53 volunteers aged 22 to 39 years (34 males and 19 females, mean age: 26.0 years) who provided informed consent before the study (Table 1). Individuals with a history or current diagnosis of periodontal disease, periapical periodontitis in the mandible, or biological diseases were excluded. This study was approved by the Ethics Committee of Matsumoto Dental University (Approval No. 0342). All participants provided informed consent, and privacy was maintained throughout this study. To ensure confidentiality, all data collected were anonymized. Participants were informed of their right to participate voluntarily and that they could withdraw from the study at any time without consequence.

**Table 1:** Characteristics of the study participants al-BMD: alveolar bone mineral density, ROI: region of interest, LSBMD: lumbar spine bone mineral density, FNBMD: femoral neck bone mineral density, SD: standard deviation

Variables	Age (years)	Body Height (cm)	Body Weight (kg)	al-BMD (mg/cm²)			LSBMD (g/cm²)	FNBMD (g/cm²)
				ROI 1	ROI 2	ROI 3		
Mean (SD)	25.96 (3.77)	168.10 (8.89)	66.44 (16.04)	1141.17 (292.90)	1273.46 (324.32)	1417.74 (383.97)	1.23 (0.14)	1.01 (0.14)

**Figure 1:**
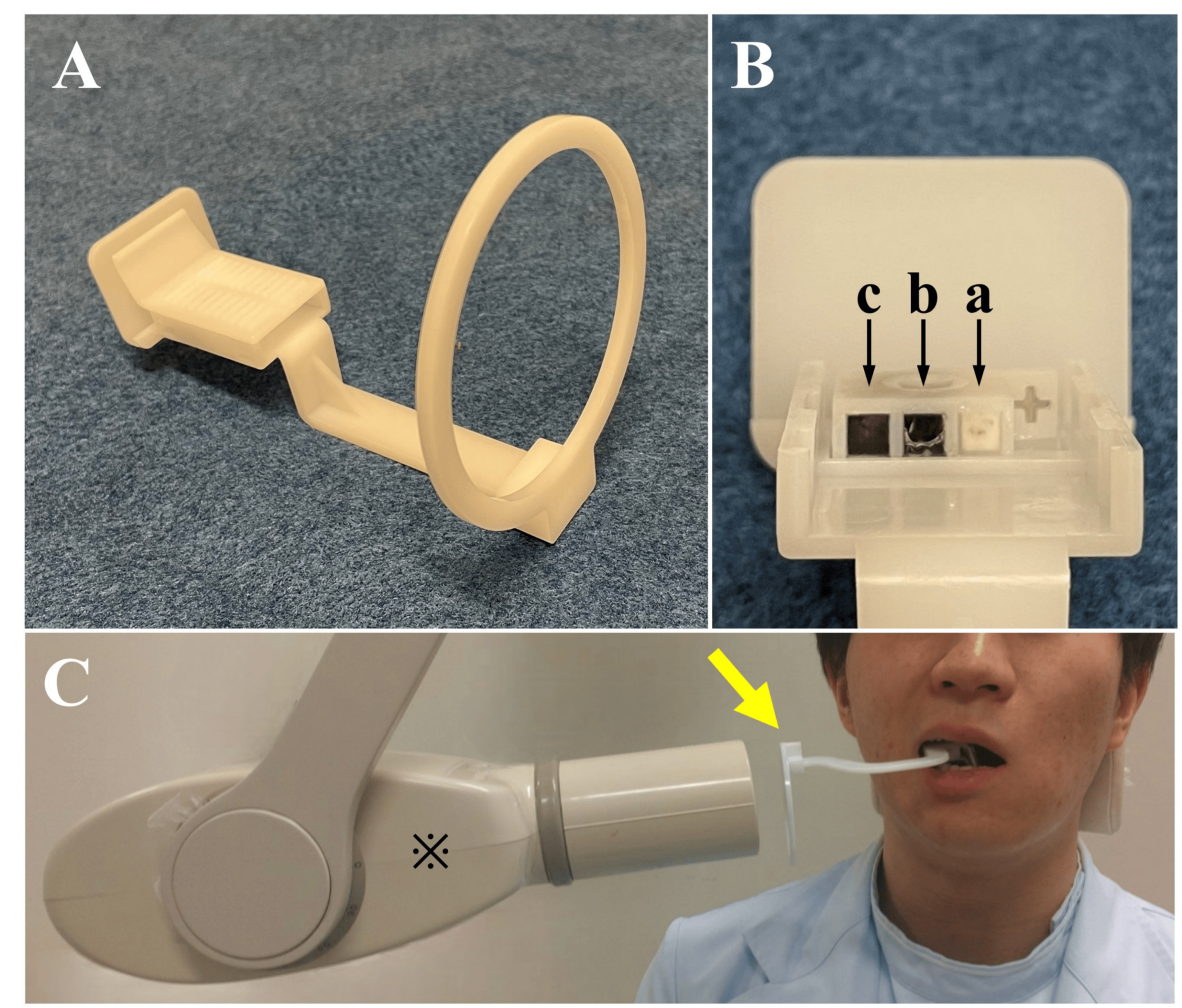
A specialized indicator A specialized indicator (A) was employed during imaging and contains calcium carbonate reference standards (B) for alveolar bone mineral density (al-BMD) (20%: 500 mg/cm³ (a), 60%: 1000 mg/cm³ (b), and 100%: 1500 mg/cm³ (c)). (C) shows intraoral radiography (※: X-ray tubehead) of the mandibular premolar region performed using a specialized indicator (yellow arrow).

**Figure 2:**
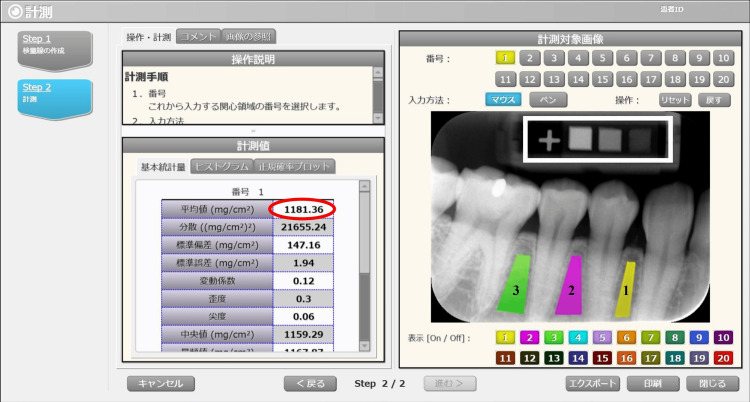
DentalSCOPE DentalSCOPE automatically compared the density of the reference standards (white rectangle) with the regions of interest (ROIs) designated in the images (ROI 1: yellow, ROI 2: purple, and ROI 3: green) and quantified the alveolar bone mineral density (al-BMD) (red circle).

Intraoral radiographs were obtained using the Max-F1 intraoral X-ray system (J. Morita, Kyoto, Japan) with an imaging plate (IP) detector under our hospital's protocol (tube voltage: 59.8 kV, tube current: 10 mA). A specialized indicator containing calcium carbonate reference standards for al-BMD (20%: 500 mg/cm³, 60%: 1000 mg/cm³, and 100%: 1500 mg/cm³) was employed during imaging (Figure 1). The imaging area was set to the mandibular premolar region, as it has sufficient buccolingual bone width compared to the anterior region and is less affected by occlusion than the molar region [6]. The captured IPs were scanned using the Digora Optime system (J. Morita, Kyoto, Japan), and the images were output in JPEG format. The image data were processed using DentalSCOPE (Media Co., Tokyo, Japan), an AI-powered software designed for al-BMD analysis. This software is based on microdensitometry (MD) techniques. When intraoral radiographic images captured together with the reference standards are input into the software, a calibration curve is automatically generated based on the image density and the BMD of the reference standards. Subsequently, it compared the density of the reference standards with the regions of interest (ROIs) designated in the images and quantified al-BMD (Figure 2). In this study, ROIs were set as follows: between the canine and the first premolar (ROI 1), between the first and second premolars (ROI 2), and between the second premolar and the first molar (ROI 3).

**Figure 3:**
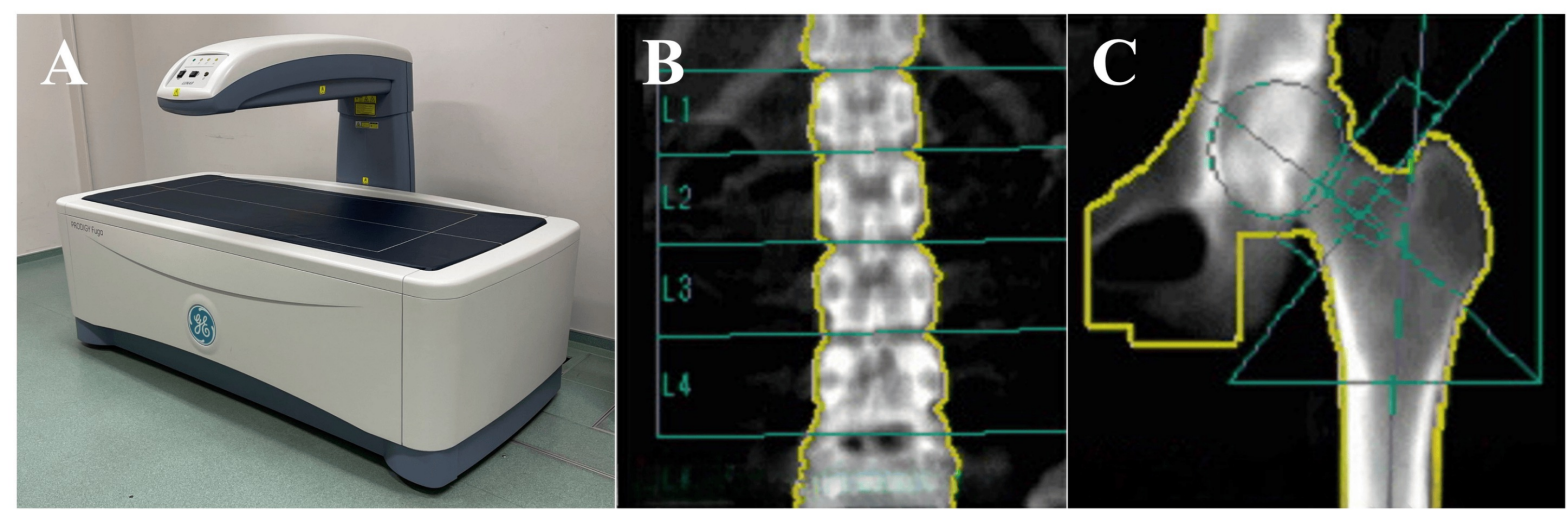
DXA and BMD analysis Dual-energy X-ray absorptiometry (DXA) (A) was used to measure lumbar spine bone mineral density (LSBMD) (B) and femoral neck bone mineral density (FNBMD) (C).

The gs-BMD was measured using dual-energy X-ray absorptiometry (DXA) with a Lunar iDXA device (GE Healthcare, Tokyo, Japan) at the lumbar spine and femoral neck. The scanning conditions were consistent with our hospital's protocol: tube voltage and tube current were set to 76.0 kV and 3.0 mA for the lumbar spine, and 76.0 kV and 0.75 mA for the femoral neck. The DXA measurements were conducted to obtain BMD values of these regions for the purpose of analyzing their correlation with al-BMD and were not interpreted using clinical thresholds or diagnostic criteria such as T-scores (Figure 3).

The sex differences between al-BMD and DXA measurements were evaluated using independent t-tests. The correlations among age, body height, body weight, al-BMD, and DXA measurements were analyzed using Peason’s correlation analysis. The correlations between al-BMD and DXA measurements were also analyzed using Peason’s correlation analysis. Statistical analyses were performed using SPSS Statistics version 19 (IBM Corp., Armonk, NY, USA), with the level of significance set at 5%.

## Results

No significant differences were observed between sexes in age, body height, body weight, al-BMD, or DXA measurements, including ROI 1-3 (Table 2). Lumbar spine bone mineral density (LSBMD) was 1.24±0.15 g/cm² in men and 1.22±0.12 g/cm² in women (P = 0.20) while femoral neck bone mineral density (FNBMD) was 1.03±0.15 g/cm² in men and 1.00±0.12 g/cm² in women (P = 0.11). Both showed no statistically significant differences between the sexes.

**Table 2:** Comparison of age, body height, body weight, al-BMD, and DXA measurements between men and women al-BMD: alveolar bone mineral density, ROI: region of interest, LSBMD: lumbar spine bone mineral density, FNBMD: femoral neck bone mineral density

Variables		Men (n=34)	Women (n=19)	Total (n=53)	p-value
Age (years)		26.04±3.53	25.83±4.26	25.96±0.52	0.67
Body Height (cm)		173.30±5.10	158.789±6.15	168.10±1.22	0.48
Body Weight (kg)		73.52±13.72	53.789±11.61	66.44±2.20	0.36
al-BMD (mg/cm²)	ROI 1	1150.87±318.25	1120.01±240.87	1141.17±49.51	0.37
	ROI 2	1300.21±366.67	1224.68±230.15	1273.46±46.81	0.23
	ROI 3	1475.49±419.75	1313.07±292.31	1417.74±57.24	0.83
LSBMD (g/cm²)		1.24±0.15	1.22±0.12	1.23±0.02	0.20
FNBMD (g/cm²)		1.03±0.15	1.00±0.12	1.01±0.02	0.11

Age tended to be correlated with FNBMD (P = 0.084) (Table 3). A significant positive correlation was observed between body weight and LSBMD (r = 0.437, P = 0.001). Conversely, a significant negative correlation was identified between body weight and FNBMD (r = -0.412, P = 0.002). Furthermore, there were no significant correlations between ROI 1-3 and LSBMD or FNBMD (Table 4).

**Table 3:** Correlation between age, body height, body weight, and BMD al-BMD: alveolar bone mineral density, ROI: region of interest, LSBMD: lumbar spine bone mineral density, FNBMD: femoral neck bone mineral density

Variables	al-BMD			LSBMD	FNBMD
	ROI 1	ROI 2	ROI 3		
	Correlation Coefficients (p-value)				
Age	-0.070 (P=0.691)	-0.057 (P=0.703)	-0.062 (P=0.684)	0.050 (P=0.721)	-0.242 (P=0.084)
Body Height	0.033 (P=0.853)	0.069 (P=0.643)	0.069 (P=0.653)	0.148 (P=0.292)	-0.116 (P=0.413)
Body Weight	-0.136 (P=0.436)	-0.059 (P=0.691)	0.069 (P=0.653)	0.437 (P=0.001)	-0.412 (P=0.002)

**Table 4:** Correlation between al-BMD and gs-BMD al-BMD: alveolar bone mineral density, gs-BMD: general skeletal bone mineral density, ROI: region of interest, LSBMD: lumbar spine bone mineral density, FNBMD: femoral neck bone mineral density

Variables	LSBMD	FNBMD
	Correlation Coefficients (p-value)	
ROI 1	-0.162 (P=0.353)	-0.220 (P=0.212)
ROI 2	-0.145 (P=0.326)	-0.181 (P=0.223)
ROI 3	-0.187 (P=0.222)	-0.178 (P=0.247)

## Discussion

In this study, no correlations were observed between age and al-BMD or LSBMD while age tended to be correlated with only FNBMD. It is possible that the relatively younger age of the participants may contribute to these results. The minimal age-related changes may contribute to no correlation between al-BMD and LSBMD. Previous studies have reported that al-BMD increased or remained unchanged from 25 to 49 years although this decreased after the age of 50 years [7,8]. Peak LSBMD typically was found around the age of 30 years, whereas FNBMD rapidly increased and showed maximum at approximately 18-20 years of age [9-11]. FNBMD declines after that and its rate of decrease after the age of 20 is greater than that of LSBMD [10,12]. These findings suggest that age-related declines in BMD may occur earlier in the femoral neck compared to the alveolar bone or lumbar spine. The results of this study imply the possibility that age-related changes in BMD might be different across these regions. Thus, al-BMD does not necessarily be associated with gs-BMD changes. In addition, alveolar bone is strongly influenced by oral health as well as local factors such as inflammation and occlusion [4]. This may contribute to the al-BMD fragility even in the case of normal gs-BMD. From the viewpoint of these results, it is better for us to evaluate the al-BMD independent of gs-BMD.

The al-BMD was not associated with body weight and body height. Alveolar bone is influenced by occlusal forces and local environmental factors [4]; however, the influence of body weight and body height against al-BMD remains uncertain and warrants further research. Conversely, LSBMD and FNBMD were influenced by body weight and body height. Body weight significantly correlated with LSBMD (r = 0.437, P = 0.001), whereas a significant negative correlation was observed between body weight and FNBMD (r = -0.429, P = 0.002). Previous reports suggest that an increase in body weight may contribute to increased LSBMD [13,14]. However, a negative correlation was found between body weight and FNBMD in this study. This means that increased body weight was associated with reduced FNBMD. Some studies suggest that an increase in body fat may be associated with decreased FNBMD [15,16]. Body height, however, was not significantly correlated with al-BMD, LSBMD, or FNBMD in this study.

This study has some limitations. Age tended to be correlated with FNBMD, although this did not reach significance. It is possible that the small sample size may influence this result. Additionally, several factors, such as oral health status, local environmental conditions, and occlusal forces, which may influence al-BMD, were not examined in this study. The gs-BMD also is influenced by lifestyle factors such as smoking, alcohol consumption, physical activity, and diet (particularly calcium and vitamin D intake). Future studies with larger sample sizes and detailed consideration of factors influencing BMD would be necessary to validate and expand upon these findings. Furthermore, this study focused on measuring LSBMD and FNBMD, as these sites are considered clinically significant for osteoporosis screening and diagnosis [17,18]. However, it is well-known that osteoporosis also affects other skeletal sites such as the distal radius of the wrist [19]. Therefore, future studies should consider incorporating distal radius BMD measurements to provide a more comprehensive evaluation of overall skeletal health. In addition, this study primarily considered body height and body weight as biological factors influencing BMD. However, other parameters, such as physical activity levels, dietary intake (e.g., calcium and vitamin D), and hormonal factors, may also play significant roles in bone health. Future studies should aim to include a broader range of factors to provide a more comprehensive understanding of BMD determinants.

## Conclusions

This study examined the relationship between al-BMD and gs-BMD (lumbar spine and femoral neck). Age-related changes in al-BMD and LSBMD were relatively stable in our study, whereas FNBMD showed a tendency to decrease with increasing age. The al-BMD was not correlated with both body weight and body height although LSBMD and FNBMD were significantly correlated with them. The factors associated with al-BMD may be different from those with gs-BMD.
